# Poor semen parameters are associated with abnormal methylation of imprinted genes in sperm DNA

**DOI:** 10.1186/s12958-022-01028-8

**Published:** 2022-11-10

**Authors:** Bing Song, Yujie Chen, Chao Wang, Guanjian Li, Zhaolian Wei, Xiaojin He, Yunxia Cao

**Affiliations:** 1 Reproductive Medicine Center, Department of Obstetrics and Gynecology, the First Affiliated Hospital of Anhui Medical University, 230032 Hefei, China; 2 NHC Key Laboratory of Study on Abnormal Gametes and Reproductive Tract, Anhui Medical University, 230032 Hefei, China; 3 Ministry of Education Key Laboratory of Population Health Across Life Cycle, Anhui Medical University, 230032 Hefei, China; 4 Anhui Province Key Laboratory of Reproductive Health and Genetics, 230032 Hefei, China; 5 Biopreservation and Artificial Organs, Anhui Provincial Engineering Research Center, Anhui Medical University, 230032 Hefei, China; 6 Department of Gynecology and Obstetrics, The Affiliated Wuxi Maternity and Child Health Care Hospital of Nanjing Medical University, 214000 Wuxi, China

**Keywords:** DNA methylation, Epigenetics, Infertility, Sperm DNA damage

## Abstract

**Background:**

Altered sperm DNA methylation patterns of imprinted genes as well as certain spermatogenesis-related genes has been proposed as a possible mechanism of male subfertility. Some reports suggest that there is an elevated risk of congenital diseases, associated with imprinted genes, in children conceived via intra-cytoplasmic sperm injection, due to the involvement of spermatozoa with aberrant imprinted genes obtained from infertile men.

**Methods:**

In this study, the DNA methylation status of the promoter regions of six imprinted genes, namely potassium voltage-gated channel subfamily Q member 1 (*KCNQ1*), maternally expressed gene 3 (*MEG3*), insulin-like growth factor 2 (*IGF-2*), KCNQ1 overlapping transcript 1 (*KCNQ1OT1*), mesoderm specific transcript (*MEST*), and paternally expressed gene 3 (*PEG3*), were detected by a next generation sequencing-based multiple methylation-specific polymerase chain reaction analysis of sperm samples obtained from 166 men who sought fertility evaluation in our Reproductive Medicine Center. Thereafter, the semen samples were classified into subgroups according to sperm motility and DNA integrity status.

**Results:**

As compared to the normozoospermic group, the samples of the asthenospermic group exhibited significant hypermethylation in two CpG sites of *IGF-2* and significant hypomethylation in one CpG site of *KCNQ1* as well as three CpG sites of *MEST* (*P* < 0.05). However, we did not observe any significant differences in the overall methylation levels of these six imprinted genes (*P* > 0.05). Additionally, we found that 111 of 323 CpG sites were hypomethylated in the group with DNA fragmentation index (DFI) ≥ 30% as compared to the group with DFI < 30% (*P* < 0.05). In this case, there were significant differences in the overall methylation levels of *MEG3*, *IGF-2*, *MEST*, and *PEG3* (*P* < 0.05), but not in that of *KCNQ1OT1* and *KCNQ1* (*P* > 0.05). Hence, aberrant methylation patterns of imprinted genes were more prevalent in males with poor sperm quality, especially in those with severe sperm DNA damage.

**Conclusion:**

In conclusion, abnormal DNA methylation of some CpG sites of imprinted genes are associated with poor sperm quality, including asthenospermia and severe sperm DNA impairment.

**Supplementary Information:**

The online version contains supplementary material available at 10.1186/s12958-022-01028-8.

## Background

During spermatogenesis, as the sperm cells mature, they undergo a series of specific epigenomic modifications to ensure proper chromatin condensation [1]. The gamete-imprinted genes are crucial for embryonic as well as placental development and growth [2]. Previously, some studies have demonstrated that acquired traits in male mice can induce epigenetic changes in their sperms, which, in turn, can influence their offspring’s physiology [3, 4]. In fact, there are some reports suggesting that children conceived via assisted reproductive technologies (ART) have an elevated risk of suffering from congenital imprinting diseases [5–7], and this might be due to the aberrant imprinting in spermatozoa of infertile men [8]. Therefore, it has been proposed that the semen quality of patients with idiopathic infertility is associated with abnormal DNA methylation of imprinted genes.

Previously, several studies have focused on the role of epigenetics in spermatogenesis and male infertility [9–11], especially the relationship between improper DNA methylation of imprinted genes in sperms and semen parameters [11–13]. Till date, several genes carrying defective methylations have been associated with poor semen parameters and population with reduced fertility. Two such genes that have been studied the most are the paternally imprinted *H19* gene and the maternally imprinted mesoderm specific transcript (*MEST*) gene [10, 14, 15]. A meta-analysis reported that as compared to the *H19* methylations in the sperm DNAs of fertile men, there was almost ten-fold higher risk ratio of aberrant *H19* methylation in the sperm DNAs of infertile men [16]. Likewise, methylation defects in three maternally imprinted genes have been identified in sperm DNAs of oligospermia patients [17–20]. Additionally, hypermethylation of *MEST*, paternally expressed gene 3 (*PEG3*), and zinc finger protein which regulates apoptosis and cell cycle arrest (*ZAC*) genes has been detected in spermatozoa of male partners from couples experiencing recurrent pregnancy loss [21]. Indeed, the improper methylation of the paternally imprinted potassium voltage-gated channel subfamily Q member 1 (*KCNQ1*) gene, insulin-like growth factor 2 (*IGF-2*) gene and KCNQ1 overlapping transcript 1 (*KCNQ1OT1*) genes have been found to be associated with oligozoospermia and asthenospermia in infertile males [22]. Incidentally, imprinting errors may be related to the sperm DNA integrity status. *KCNQ1* and *IGF-2* have also been documented to be associated with serious sperm DNA damage in infertile males [23].

Interestingly, our previous study has shown that imprinting genes are associated with relatively higher rates of differentially methylated CpG sites than non-imprinting genes in case of patients with sperm DNA damage [12]. Until now, studies have largely focused on the analysis of only a few imprinted genes and a small fraction of genomic CpG sites. Hence, in this study, we aimed to investigate the correlation between poor semen parameters and the DNA methylation status of the promoter regions ofcandidate imprinted genes in human semen samples. The DNA methylation status of CpG sites within the promoter regions of six imprinted genes, namely *KCNQ1*, maternally expressed 3 (*MEG3*), *IGF-2*, *KCNQ1OT1*, *MEST*, and *PEG3*, were examined by bisulfite sequencing of sperm DNAs obtained from 166 men.

## Materials and methods

### Semen samples

Semen samples were collected from 166 males who sought fertility evaluation at the Reproductive Medicine Center of the First Affiliated Hospital of Anhui Medical University between November 2017 and May 2019. Individuals with a history of heavy smoking, alcohol consumption, or direct exposure to environmental pollutants were excluded from the study. This study was approved by the Ethical Review Board of The First Affiliated Hospital of Anhui Medical University.

### Semen parameters analysis by computer-assisted sperm analysis

All semen samples were collected by masturbation into sterile containers after 3–7 days of sexual abstinence. Conventional semen parameters, including semen volume, sperm concentration, and progressive motility rate (PR), were determined using computer-assisted sperm analysis (CASA), according to the World Health Organization guidelines [24]. For this study, only the semen samples with the following characteristics were selected: semen volume ≥ 1.5mL, sperm concentrations ≥ 15 × 10^6^ cells/mL, PR ≥ 10%, and white blood cell count ≤ 10^6^ cells/mL, as observed by light microscopy. A total of 74 men with mild-to-moderate asthenozoospermia (PR, 10–32%; asthenozoospermia group) and 92 with normal semen characteristics (normozoospermia group) were included.

### Measurement of DNA fragmentation using sperm chromatin structure assay

The DNA integrity status of the sperm samples was assessed by sperm chromatin structure assay (SCSA), following the manufacturer’s recommendations [25]. Initially, the sperm samples were exposed to an acidic detergent solution, followed by an acridine orange staining. The fluorescence patterns were assessed using a BD FACScan flow cytometer (Becton Dickinson, San Jose, CA, USA). Red fluorescence indicated single or double strand breaks of DNA and green fluorescence represented normal intact DNA. Sperm DNA Fragmentation index (DFI) of the sperm samples can be calculated based on the ratio of red to total fluorescence intensity. Subsequently, the samples were also divided into two groups according to sperm DFI, namely low DFI group (DFI ≤ 30%) and severely sperm DNA impaired group (DFI > 30%).

### Sperm DNA isolation and sodium bisulfite treatment

We washed the semen samples twice with phosphate buffered saline to remove as many somatic cells as possible, and treated the semen samples by density gradient centrifugation. Each sample was then tested for round-cell contamination and, if somatic contamination was identified, the entire sample was treated with swimming- up techniques prior to genomic DNA isolation. Genomic DNA was then extracted by the commercial DNA kit (Qiagen Inc., Germany), according to protocol of the manufacturer. Nanodrop 2000 (Thermo Scientific) was used to estimate the purity and concentration of the isolated DNA. Subsequently, 500 ng of genomic DNA from each sample was treated with sodium bisulfite using the EZ DNA methylation-Gold Kit (Zymo Research, USA), following standard protocol of the manufacturer.

### DNA methylation analysis

The methylation status of the promoter regions of the six selected imprinted genes (*KCNQ1*, *MEG3*, *IGF-2*, *KCNQ1OT1*, *MEST* and *PEG3*) in the sperm DNA was detected by a next generation sequencing (NGS)-based multiple methylation-specific polymerase chain reaction (PCR) analysis (MethylTarget™, Genesky Biotechnologies Inc., Shanghai, China). We analyzed a total of 323 CpG sites by the targeted bisulfite sequencing, following the manufacturer’s instructions.The information of the six genes and CpG sites were shown in Table 1. Multiplex PCR was executed with primers projected by MethPrimer (http://www.urogene.org/) to observe the transformed sequences (Table S1). The PCR amplification and the high-throughput bisulfite sequencing with the Illumina MiSeq sequencing platform (Illumina, San Diego, CA, USA) were carried out, following our previously depicted protocols.The data obtained after sequencing were prepared, filtered, and aligned applying the BiQ Analyzer HT software, as stated by protocols of the producer.

**Table 1:** The information of CpG sites for the 6 selected genes

Gene	Chr	CpG islands	Detected CpG sites	Paternal/Maternal imprinted
*KCNQ1*	11	1	27	paternal
*MEG3*	14	3	35	paternal
*IGF-2*	11	4	105	maternal
*KCNQ1OT1*	11	2	41	maternal
*MEST*	7	5	96	maternal
*PEG3*	19	1	19	maternal

Chr: Chromosome

### Statistical analysis

SPSS software (version 22.0; SPSS Inc., USA) was employed to analyze the collected data. The Mann-Whitney test, parametric, and nonparametric tests were used to describe the test results following assessment of the normal distribution. The results of the methylation analysis are expressed as mean ± standard deviation (S.D.). Statistical significance was set at *P* < 0.05.

## Results

### Clinical data

The semen parameters and clinical features of the 166 men participating in this study are listed in Table 2. There were no statistically significant differences between the mean ages of the men included in the asthenospermia and normozoospermia groups (31.35 ± 4.35 y and 31.68 ± 3.64 y, respectively; *P* > 0.05). Moreover, the semen volume, sperm concentration, and DFI were also similar for both the groups, with no significant differences (*P* > 0.05). The mean PR of sperms in the samples of the asthenospermia group (23.79 ± 6.69%) was significantly lower (*P* < 0.01) than that in the normozoospermia group (51.62 ± 11.82%). Interestingly, when these 166 semen samples were classified according to their DFI values into either the sperm DNA damage group or the normal group, there were no significant differences with respect to the age of the men, their semen volume, sperm concentration, and PR rate. The DFI was significantly higher (*P* < 0.01) in the DNA damage group (37.03 ± 6.39%) as compared to that of the normal group (13.42 ± 5.47%).

**Table 2:** Clinical characteristics and semen parameters in the study

Items	Normozoospermia (n = 92)	Asthenospermia (n = 74)	*P*	DFI ≤ 30% (n = 119)	DFI > 30%(n = 47)	*P*
Age(years)	31.68 ± 3.64	31.35 ± 4.35	NS	31.28 ± 3.88	32.19 ± 4.14	NS
Semen volume(ml)	3.946 ± 1.38	3.796 ± 0.17	NS	3.80 ± 1.29	4.09 ± 1.67	NS
Sperm concentration (*10^6/mL)	92.51 ± 71.06	75.29 ± 57.66	NS	87.87 ± 68.59	77.15 ± 58.15	NS
Progressive Motility(%)	51.62 ± 11.82	23.79 ± 6.69	< 0.01	42.36 ± 18.50	31.25 ± 8.12	NS
DFI(%)	18.78 ± 10.50	21.38 ± 13.32	NS	13.42 ± 5.47	37.03 ± 6.39	< 0.01

DFI, DNA fragmentation index; NS, not significant. Patients clinical characteristics are expressed as mean and standard deviations and analysed by t test

### The CpG site methylation of imprinted genes in asthenospermia patients

We assessed the DNA methylation patterns of the selected gene regions using the MethylTarget technique, and detected 323 CpG sites, of which only 6 (2 in *IGF-2*, 1 in *KCNQ1*, and 3 in *MEST*) were significantly different between the mild asthenospermia and normozoospermia groups (Table 3**)**. Compared to the methylations detected in the CpG sites of the *IGF-2* in the normozoospermia group, four sites showed hypomethylation and two sites showed hypermethylation in the corresponding regions of the asthenospermia group. On the contrary, analysis of the promoter regions of the other three genes (*MEG3*, *KCNQ1OT1*, and *PEG3*) indicated that there were no significant differences in DNA methylation between the two groups.

**Table 3:** The DNA Methylation levels of selected candidate genes in the mild asthenospermia group and normozoospermia group

Gene	Position	Case Value	Control Value	*P*
*IGF-2*	169	0.014 ± 0.007	0.011 ± 0.006	**0.025**
*IGF-2*	55	0.007 ± 0.004	0.006 ± 0.003	**0.018**
*KCNQ1*	29	0.004 ± 0.006	0.007 ± 0.009	**0.017**
*MEST*	37	0.012 ± 0.006	0.014 ± 0.007	**0.035**
*MEST*	58	0.007 ± 0.004	0.009 ± 0.007	**0.028**
*MEST*	186	0.007 ± 0.007	0.011 ± 0.010	**0.004**

Case:asthenospermia group; Control:normozoospermia group; Bold values was statistically significant (*P* < 0.05)

### The CpG site methylation of imprinted genes in patients with severely impaired sperm DNA

Among the 323 detected CpG sites, the methylation levels of 111 CpG sites in 5 genes (*MEG3*, *IGF-2*, *KCNQ1OT1*, *MEST*, and *PEG3*) were significantly different between the individuals with low DFI group and those with severely impaired sperm DNA (Table 4, *P* < 0.05). No significant differences were found in the remaining 212 CpG sites. The average rate of differentially methylated positions (DMPs) was 34.37%. The three genes with the highest DMP percentages were *PEG3*, *MEG3*, and *KCNQ1OT1* (100%, 62.9%, and 51.2%, respectively). The average value of all CpG sites in the gene promoter regions was considered as the overall methylation value of each gene. Notably, *IGF-2*, *MEG3*, *MEST*, and *PEG3* showed significant differences in the overall methylation levels among the detected genes (*P* < 0.05).

**Table 4:** The DNA Methylation levels of selected candidate genes in patients with severely impaired sperm DNA.

Gene	CpG Sites	DMP	DMP(%)	Case Value	Control Value	*P* Value
*IGF-2*	105	16	15.2	0.009 ± 0.002	0.010 ± 0.004	**0.029**
*KCNQ1*	27	0	0	0.007 ± 0.002	0.007 ± 0.002	0.321
*KCNQ1OT1*	41	21	51.2	0.040 ± 0.036	0.055 ± 0.064	0.066
*MEG3*	35	22	62.9	0.027 ± 0.027	0.043 ± 0.066	**0.026**
*MEST*	96	33	34.4	0.030 ± 0.031	0.045 ± 0.064	**0.043**
*PEG3*	19	19	100	0.027 ± 0.029	0.045 ± 0.063	**0.014**

Case:severely impaired sperm DNA group (DFI > 30%); Control:low DFI group(DFI ≤ 30%); Bold values was statistically significant (*P* < 0.05)

## Discussion

The methylation status of a quantity of imprinted genes of the sperm DNA has been investigated concerning reproductive dysfunction and/or impaired spermatogenesis in males [26]. In fact, imbalances in the methylation patterns have been detected in case of both paternally and maternally imprinted genes in the sperm DNA derived from infertile males with abnormal semen parameters [10]. Therefore, the role of DNA methylation in male infertility and spermatogenesis deserves further attention [27].

In this study, we examined the DNA methylation status of CpG sites in the promoter regions of two paternally imprinted genes, namely *KCNQ1* and *MEG3*, and four maternally imprinted genes, namely *IGF-2*, *KCNQ1OT1*, *MEST*, and *PEG3*, by employing deep bisulfite sequencing of the target genes. First, we analyzed the differential methylation between sperm DNA samples obtained from males with mild to moderate asthenospermia and normozoospermia. Only 6 out of 323 detected CpG sites were found to be significantly different between the two groups. We identified CpG sites associated with three imprinted genes, namely *IGF-2, MEST*, and *KCNQ1*, displaying aberrant methylation, but there were no alterations in the imprinted genes *MEG3*, *KCNQ1OT1*, and *PEG3*. Our results suggest that DNA methylation status may be associated with the poor semen parameters in infertile males. This hypothesis was further confirmed in the subsequent analysis. We observed that aberrant methylation patterns of imprinted genes were more prevalent in patients with severely impaired sperm DNA intergrity status. The imprinted genes *PEG3*, *MEG3*, and *KCNQ1OT1* presented a very high proportion of differential methylation, each with > 50% DMPs in the detected CpG sites. Although there have been indications that the spermatozoa of infertile males can carry imprinting defects, a relatively small sample size patient cohort was investigated. Generally, our results are in accord with preceding studies that show that sperm DNA methylation patterns are correlated with sperm quality parameters [11–20].

Abnormal genomic imprinting may lead to inappropriate gene transcription or gene repression. The accurate expression of imprinted genes is responsible for embryo development, fetal growth, and behavioral syndromes [28]. Moreover, the aberrant DNA methylation status of the imprinted genes of sperm can be transmitted directly to the embryo, as imprinted genes can escape epigenetic reprogramming after fertilization [29, 30]. It has been widely reported that children conceived through ART have a higher incidence of imprinting-related genetic diseases than those conceived naturally, and this might be due to the epigenetic errors in gametes [6, 7]. Growing evidence were confirmed that the paternal epigenome plays a crucial role during embryo development [30].The results of our study may raise certain concerns about the risk of patients with poor semen parameters, especially severe impairments in the sperm DNA, transmitting epigenetic dysfunctions to their offsprings.

Although many genetic and environmental factors have been confirmed to affect semen quality parameters, the vast majority of male infertility is still unknown [30–32]. The DNA methylation status of sperms has emerged as a novel biomarker that can provide information related to male fertility [27, 33, 34]. Incidentally, oxidative stress commonly causes several conditions that are associated with male infertility [35]. High levels of reactive oxygen species (ROS) impair sperm quality by decreasing sperm motility and inducing sperm DNA damage [36]. Multi-antioxidant supplementation is considered effective for the improvement of male fertility parameters, since they lead to a decrease in the ROS concentration [37]. Previously, a study has reported that semen quality is negatively correlated with ROS levels [38]. Interestingly, excessive levels of ROS adversely alter DNA methylation, thereby leading to epigenetic errors [39]. Oxidative stress is associated with global hypomethylation and aberrant hypermethylation of target gene promoter regions [40]. One possible mechanism is that ROS causes an upregulated expression of DNA methyltransferases (DNMTs) or the formation of a new DNMT containing complex that results in site-specific hypermethylation. Another explanation is that ROS yield raises DNA oxidation structures’ formation, such as 8-hydroxy-2’-deoxyguanosine and 5-hydroxymethylcytosine, both of which can induce global DNA hypomethylation through suppressing DNA methylation at neighboring cytosine bases [9, 36, 41]. Therefore, we inferred that the abnormal DNA methylation patterns in patients with poor semen parameters might be a result of ROS-induced alterations in DNA methylation. Imprinting genes may be more susceptible to the ROS-induced modulation of sperm DNA methylation, which, in turn, may increase the epigenetic risk of male infertility.

## Conclusion

In conclusion, our study suggests that in infertile men, the poor semen parameter, especially the severe impairments in the sperm DNA integrity, are associated with abnormal DNA methylation of both paternally and maternally imprinted genes. Furthermore, these aberrant DNA methylation patterns in the imprinted genes have the potential to be employed as useful tools in the diagnostic and prognostic setup of male infertility. Hence, further investigations are necessary to successfully implement this.

## Electronic Supplementary Material

Below is the link to the electronic supplementary material.

Supplementary Material 1

## Data Availability

The datasets used and/or analyzed during the current study are also available from the corresponding author on reasonable request.

## Abbreviations

ART: Assisted reproductive technique
MEST: Mesoderm specific transcript
PEG3: Paternally expressed gene 3
MEG3: Maternally expressed gene 3
IGF-2: Insulin-like growth factor 2
KCNQ1: Potassium voltage-gated channel subfamily Q member 1
KCNQ1OT1: KCNQ1 overlapping transcript 1
ZAC: Zinc finger protein which regulates apoptosis and cell cycle arrest
DMR: Differentially methylated region
DMP: Differentially methylated point
PR: Progressive motility
CASA: Computer-assisted sperm analysis
SCSA: Sperm chromatin structure assay
DFI: DNA Fragmentation Index
NGS: Next generation sequencing
PCR: Polymerase chain reaction
ROS: Reactive oxygen species
DNMT: DNA methyl transferase
