# Supplementary material for: Poor semen parameters are associated with abnormal methylation of imprinted genes in sperm DNA
Source: Reprod Biol Endocrinol. 2022 Nov 10;20:155. doi: 10.1186/s12958-022-01028-8 (PMC9647922; doi:10.1186/s12958-022-01028-8)
Supplement: Supplementary file 1 — Supplementary Material 1 [file 12958_2022_1028_MOESM1_ESM.docx]

Table S1.The primer sequences used for DNA methylation analyses

| Primer Name |  | Primer |
| --- | --- | --- |
| *KCNQ1* | Forward | GTTTTTAGTTGTTTTYGATTTTGGTTY |
|  | Reverse | CCCACACCAACTCTCAAAAA |
| *MEG3* | Forward | GTTAGGTGTGGGATTTGYGTTTY |
|  | Reverse | CCCTAACCRCCATAACCAACA |
|  | Forward | GGTTAATTATTTTTAGAGAAATGAGYGTATTGTAG |
|  | Reverse | TAAACRCCCCAACTCTCCTAAAAA |
|  | Forward | TGGTAGGGATTTTAGTTTAGAGGAG |
|  | Reverse | CCAAAAACCTAACCAACAACTAAC |
| *IGF2* | Forward | TTGGAGAGTTTGAAYGATGTAAGAAAG |
|  | Reverse | CTAACCCRCRAACCTAAAACTCC |
|  | Forward | GGGGTTGGGGAAGTTGTAG |
|  | Reverse | TTCTCTCATCCCCCAAACCT |
|  | Forward | ATTTGGGTTAGGTTTGGAGTTT |
|  | Reverse | ATTTGGGTTAGGTTTGGAGTTT |
|  | Forward | CCCAAACCCCCAAATTATCRT |
|  | Reverse | GGGGGTTTTTGTTGGTTGTT |
|  | Forward | ACCTCCRTAAACTCCAAACCTAACC |
|  | Reverse | TTYGTTTGATTGTTTAGGGAGGA |
|  | Forward | CTTTCTATTTCTCTCCRTACTATTCTCTCC |
|  | Reverse | GGGYGGAGTGGAGGTTGT |
|  | Forward | CCTCRAACCRCTCCCCTTTC |
| *KCNQ1OT1* | Reverse | GTTGAGGAGAGTTTGGGAGAA |
|  | Forward | AAAACTCCTCAACATAATTCTCCTC |
|  | Reverse | GTTGAATTGGGATTGGAGTTTG |
|  | Forward | TCAAACAAACTCCCAACCAAAA |
|  | Reverse | TTAGGTGGGTGGTTTGGTATG |
|  | Forward | TCCAAACCACRCCCRAAAC |
| *MEST* | Reverse | TGTGTTAGAGGTTTTGATGATAGGT |
|  | Forward | CCRATACCCRCTACATCCAAAA |
|  | Reverse | YGGTTGGGAGGGGTTTTG |
|  | Forward | GGTGTAGTTTAGGATTTTAAGATTTAGGT |
|  | Reverse | CCAACCTACCRCCCACCT |
|  | Forward | GTTTGGTAGGGAGAAGGYGGTAG |
|  | Reverse | TTCAAATAAAACCTTACCTACAAAACTCC |
|  | Forward | GTTTAGGAGGYGTGAGGTTGTG |
|  | Reverse | AATCCCCACRCCCAAAAA |
| *PEG3* | Forward | GGTGAGGTTGTTGATTGGTTAG |
|  | Reverse | ACRCACTCACCTCACCTCAATAC |
